# Updated Age- and Sex-Specific Reference Values and Anthropometric Correlates of Maximum Handgrip Strength in Preschool Children from the Araucanía Region, Chile

**DOI:** 10.3390/children13091200

**Published:** 2026-09-05

**Authors:** Andrés Godoy-Cumillaf, Josivaldo de Souza-Lima, Frano Giakoni-Ramírez, Catalina Muñoz-Strale, Maribel Parra-Saldias, Daniel Duclos-Bastias, Claudio Farias-Valenzuela, Eugenio Merellano-Navarro, José Bruneau-Chávez

**Affiliations:** 1 Grupo de Investigación en Educación Física, Salud y Calidad de Vida (EFISAL), Facultad de Educación, Universidad Autónoma de Chile, Temuco 4780000, Chile; andres.godoy@uautonoma.cl; 2 Facultad de Educación y Ciencias Sociales, Instituto del Deporte y Bienestar, Universidad Andres Bello, Las Condes, Santiago 7550000, Chile; frano.giakoni@unab.cl (F.G.-R.); catalina.munoz@unab.cl (C.M.-S.); 3 Departamento de Educación Física, Deporte y Recreación, Universidad de Atacama, Copiapó 1530000, Chile; maribel.parra@uda.cl; 4 iGEO, Escuela de Educación Física, Facultad de Filosofía y Educación, Pontificia Universidad Católica de Valparaíso, Valparaíso 2340025, Chile; daniel.duclos@pucv.cl; 5 METIS Research Lab, Facultad de Negocios y Tecnología, Universidad Alfonso X el Sabio (UAX), 28691 Madrid, Spain; 6 Escuela de Ciencias de la Actividad Física, Universidad de Las Américas, Santiago 1530000, Chile; claudio.farias.valenzuela@edu.udla.cl; 7 Department of Physical Activity Sciences, Faculty of Education Sciences, Universidad Católica del Maule, Talca 3530000, Chile; emerellano@ucm.cl; 8 Departamento de Educación Física, Deportes y Recreación, Universidad de la Frontera, Temuco 4811230, Chile; jose.bruneau@ufrontera.cl

**Keywords:** muscular fitness, physical development, pediatric assessment, health-related physical fitness, percentile curves

## Abstract

**Highlights:**

**What are the main findings?**

- Updated age- and sex-specific reference values and interpolated percentile curves for maximum handgrip strength were established for preschool children aged 4–6 years from the Araucanía Region, Chile.
- Maximum handgrip strength increased progressively with age, was consistently higher in boys than in girls, and showed the strongest associations with height and body weight.

**What are the implications of the main findings?**

- These regional reference values may provide a useful framework for describing and contextualizing handgrip strength performance among preschool children assessed under comparable conditions.
- The findings provide reference information for future research and physical fitness assessment, while nationally representative studies are needed before broader population-level or clinical applications can be established.

**Abstract:**

**Background/Objectives**: Handgrip strength is a simple and reliable indicator of muscular fitness during childhood. However, updated reference values for handgrip strength in preschool children remain limited in many countries, including Chile. This study aimed to develop updated age- and sex-specific reference values for maximum handgrip strength in preschool children aged 4–6 years from the Araucanía Region, Chile, and to examine its associations with age and anthropometric characteristics. **Methods**: This cross-sectional study included 899 preschool children (464 boys and 435 girls) from the Araucanía Region, Chile. Handgrip strength was assessed using a Takei 5401 digital dynamometer following the PREFIT protocol. Maximum handgrip strength was defined as the highest value obtained from either hand. Age- and sex-specific percentile reference values (P5, P10, P25, P50, P75, P90, and P95) and interpolated percentile curves were generated. Associations of maximum handgrip strength with age and anthropometric variables were examined using Spearman’s rank correlation coefficients. **Results**: Maximum handgrip strength increased progressively from 4 to 6 years in both sexes, with boys showing significantly higher mean values than girls at each age (all *p* ≤ 0.011; Cohen’s d = 0.28–0.65). Median (P50) maximum handgrip strength increased from 6.5 to 9.1 kg in girls and from 7.2 to 9.9 kg in boys. Height showed the strongest correlation with maximum handgrip strength (ρ = 0.554), followed by body weight (ρ = 0.463), age (ρ = 0.458), waist circumference (ρ = 0.307), and body mass index (ρ = 0.184) (all *p* < 0.001). **Conclusions**: This study provides updated age- and sex-specific reference values and interpolated percentile curves for maximum handgrip strength in preschool children aged 4–6 years from the Araucanía Region, Chile. These regional reference data may facilitate the interpretation of handgrip strength performance in preschool children assessed under comparable conditions and provide a useful resource for physical fitness assessment and future research.

## 1. Introduction

Muscular strength is increasingly recognized as a fundamental component of health-related physical fitness during childhood and adolescence, contributing to motor performance and functional capacity and showing associations with cardiometabolic, skeletal, and overall health outcomes [1,2,3,4]. However, much of the evidence linking muscular strength to clinical outcomes derives from older populations or from school-aged children and adolescents. In preschool children, overt cardiometabolic disease and age-related functional decline are uncommon; therefore, muscular strength is more appropriately considered a component of physical fitness and an indicator of physical growth and neuromuscular development rather than a direct marker of clinical disease.

Handgrip strength (HGS) is a widely used measure of muscular strength because handgrip dynamometry is inexpensive, portable, non-invasive, and easy to administer [5]. HGS has demonstrated good reliability and validity as an indicator of muscular fitness, making it particularly suitable for large-scale assessments in pediatric populations [5,6]. Although HGS has been associated with cardiometabolic risk indicators and normalized HGS thresholds have been proposed in older pediatric populations [2,3,4], these findings should not be directly extrapolated to preschool-aged children, for whom clinically validated HGS thresholds are lacking. At this age, HGS is therefore better interpreted as a practical measure of muscular fitness and physical development, while its relationship with subsequent health trajectories requires further longitudinal investigation.

The preschool years represent an important period for the assessment of muscular fitness because rapid changes in body size, neuromuscular coordination, motor competence, and physical capacity occur during this stage of development [7,8]. HGS may help characterize these developmental differences and complement broader assessments of physical fitness in young children. Nevertheless, most available age- and sex-specific HGS reference values have been developed in school-aged children and adolescents, with comparatively fewer studies focusing on children younger than seven years [9,10,11,12]. International studies from Spain, Colombia, Saudi Arabia, Peru, and other populations have also demonstrated age- and sex-related differences in HGS and substantial variation across populations [9,10,11,12]. These findings support the need for age-, sex-, and population-specific reference data to appropriately contextualize HGS performance during early childhood.

In Chile, previous research has reported age- and sex-specific reference values for several physical fitness components, including HGS, in preschool children aged 4–6 years [13]. However, HGS represented only one component of a broader physical fitness assessment, and updated HGS-specific reference values derived from a larger and geographically broader regional sample remain warranted. Furthermore, evidence regarding the associations between HGS and anthropometric characteristics during the preschool years in Chilean populations remains limited [14]. Updated regional reference data may therefore contribute to a more contextualized interpretation of muscular fitness and provide a basis for future research in Chilean preschool children.

Therefore, the primary aim of this study was to develop updated age- and sex-specific reference values for HGS in preschool children aged 4–6 years from the Araucanía Region, Chile. A secondary aim was to examine the associations between HGS and anthropometric characteristics, including body weight, height, body mass index, and waist circumference.

## 2. Materials and Methods

### 2.1. Study Design and Participants

Data were obtained from two independent cross-sectional research projects conducted in the Araucanía Region, Chile. The first project was carried out between September and November 2022. Educational establishments from 20 municipalities in the Araucanía Region were invited to participate through existing collaboration agreements with the Faculty of Education of Universidad Autónoma de Chile. Establishments from 12 municipalities accepted the invitation, with one educational center participating from each municipality: Pucón, Cunco, Victoria, Melipeuco, Loncoche, Angol, Temuco, Lautaro, Chol Chol, Curacautín, Collipulli, and Villarrica. These centers contributed an initial sample of 567 children. The second project was conducted between September and November 2025. Ten educational centers located in Temuco with existing collaboration agreements with the Faculty of Education of Universidad Autónoma de Chile were invited to participate, and all 10 accepted the invitation, contributing an initial sample of 365 children. The number of participants contributed by each municipality and educational center is provided in Supplementary Table S1.

Educational centers were therefore selected using a non-probability convenience sampling approach based on existing institutional collaboration agreements and willingness to participate. Within each participating center, all children aged 4–6 years enrolled in Transición 1 (Pre-Kindergarten), Transición 2 (Kindergarten), or the first year of primary education were invited to participate. Children aged 4–6 years with at least one valid HGS measurement were eligible for inclusion in the analytical sample. Although the geographical recruitment of educational centers differed between the two collection periods, both projects used the same participant-level recruitment approach within participating centers, whereby all eligible children were invited to participate, as well as the same assessment protocols, equipment, examiner training, and quality-control procedures.

The original pooled dataset comprised 932 children (567 assessed in 2022 and 365 in 2025). Ten children were excluded because their recorded age was outside the predefined study range of 4–6 years, and 23 were excluded because neither hand had a valid HGS measurement. Following these exclusions, the final analytical sample comprised 899 children (553 from the 2022 cohort and 346 from the 2025 cohort).

### 2.2. Anthropometric Measurements

Anthropometric measurements were obtained by trained evaluators following standardized procedures. Body weight was measured to the nearest 0.1 kg using a calibrated digital scale (Tanita, MC-780U, Tanita Corporation, Tokio, Japan), while height was measured to the nearest 0.1 cm using a portable stadiometer (Seca Model 200, seca GmbH & Co. KG, Hamburg, Germany). Children were assessed barefoot and wearing light clothing.

Body mass index (BMI) was calculated as body weight (kg) divided by height squared (m^2^). Waist circumference was measured using a non-elastic anthropometric tape (Holway, San Jose, CA, USA) at the midpoint between the lowest rib and the iliac crest with the participant standing upright. Measurements were recorded to the nearest 0.1 cm.

### 2.3. Handgrip Strength Assessment

HGS was assessed using a Takei 5401 digital hand dynamometer (Takei Scientific Instruments Co., Ltd., Niigata, Japan) following the procedures described in the PREFIT battery for preschool children [15]. Before testing, hand span was measured for each child as the distance from the tip of the thumb to the tip of the little finger with the hand opened as wide as possible. The grip span of the dynamometer was then individually adjusted according to hand size using the equation y = x/5 + 1.5, where x represents hand span (cm) and y represents the grip span (cm). This procedure was applied consistently to all participants in both study cohorts. Children performed the test in a standing position with the arm extended alongside the body without touching the trunk. Participants were instructed to squeeze the dynamometer as forcefully as possible for a few seconds while receiving standardized verbal encouragement from the evaluator.

Both hands were assessed separately. Two trials were performed for each hand, and the highest value obtained for each hand was recorded in kilograms (kg). In addition to right- and left-hand HGS, the single highest value obtained across all valid trials from either hand was defined as maximum HGS and was considered the primary outcome for the construction of reference values and percentile curves. This definition was selected to represent each child’s maximal observed grip-strength capacity independently of hand dominance and to reduce the potential influence of side-specific performance. Because this operational definition may yield values that differ from those obtained using dominant-hand strength, right-hand strength, or bilateral mean values, right- and left-hand reference percentiles are additionally provided in the Supplementary Materials to facilitate comparison with studies using alternative HGS definitions.

### 2.4. Data Processing and Quality Control

Prior to statistical analysis, the original pooled dataset was screened for eligibility, completeness, and data-entry errors. Age eligibility was defined a priori according to the study population (4–6 years); therefore, 10 children recorded as 7 years old were excluded. For HGS, recordings of 0.0 kg were considered non-valid measurements because they did not represent a completed force measurement. Participants were retained when at least one hand had a valid HGS measurement, with maximum HGS calculated from the available valid hand. Twenty-three children had 0.0 kg recorded for both hands and were therefore excluded because maximum HGS could not be determined. Thus, 33 of the original 932 records were excluded, resulting in a final analytical sample of 899 children.

Quality control was subsequently performed using the original recorded values without applying general physiological exclusion thresholds or winsorization. Unequivocal data-entry errors were treated as missing rather than used as grounds for participant exclusion. Specifically, two body-weight recordings of 1.5 kg, the two corresponding BMI values of 1.196 kg/m^2^, and two waist-circumference recordings of 6.5 cm were treated as erroneous and set to missing. Two extreme unilateral HGS recordings (53.0 and 91.0 kg) were considered non-valid measurements, while the corresponding valid contralateral measurements were retained for calculation of maximum HGS. A unilateral HGS value of 22.25 kg was retained in the primary analysis because it could not be unequivocally identified as a data-entry error; a sensitivity analysis treating this measurement as non-valid and using the valid contralateral value confirmed that its inclusion did not materially affect the study findings.

No missing values were imputed, and analyses involving variables with missing observations were performed using available-case data. No winsorization or automatic exclusion of anthropometric outliers was performed. The same data-processing and quality-control procedures were applied uniformly to the pooled dataset.

### 2.5. Ethical Considerations

The procedures followed the ethical principles of the Declaration of Helsinki for research involving human participants. The 2022 data collection was approved by the Ethics Committee of Universidad Autónoma de Chile (Approval No. CEC N°31-22), whereas the 2025 data collection was approved by the Ethics Committee of Universidad Autónoma de Chile (Approval No. CEC N°22-25).

Written informed consent was obtained from parents or legal guardians prior to participation, and verbal assent was obtained from all children before the assessments were conducted.

### 2.6. Statistical Analysis

All statistical analyses were performed using Python version 3.13.14. Data processing and percentile estimation were conducted using pandas version 2.2.3 and NumPy version 2.3.5, whereas PCHIP was implemented using SciPy version 1.17.1. Continuous variables were summarized as means and standard deviations (SD), whereas categorical variables were expressed as frequencies and percentages.

Age- and sex-specific descriptive statistics were calculated for right-hand grip strength, left-hand grip strength, and maximum HGS. The highest value obtained from either hand was defined as maximum HGS and was used for all reference value analyses.

Sex differences in maximum HGS were examined separately within each age group using two-sided Welch independent-samples *t*-tests. Mean differences were calculated as boys minus girls and are presented with 95% confidence intervals (CIs). Cohen’s d, calculated using the pooled standard deviation, was used to quantify the magnitude of the sex differences, with positive values indicating higher maximum HGS in boys; 95% CIs were also calculated for the effect-size estimates. As a complementary analysis, the overall association between sex and maximum HGS was examined using linear regression adjusted for age.

Chronological age was available in the dataset as completed years at the time of assessment. Accordingly, participants were classified according to their recorded age as 4, 5, or 6 years. More precise age information, such as age in months, decimal age, or dates of birth, was not available in the analytical dataset. Age- and sex-specific empirical percentile distributions (P5, P10, P25, P50, P75, P90, and P95) were therefore calculated separately for each of the three available age categories. Percentiles were estimated within each age-by-sex subgroup using the pandas.Series.quantile() function with its linear interpolation method (interpolation = ‘linear’), whereby percentile values falling between adjacent ordered observations were estimated by linear interpolation.

For graphical presentation, shape-preserving piecewise cubic Hermite interpolation (PCHIP) was applied separately for each sex and percentile using the empirical percentile values observed at ages 4, 5, and 6 years as interpolation knots [16]. PCHIP was implemented using scipy.interpolate.PchipInterpolator, and each curve was evaluated at 201 equally spaced points between ages 4 and 6 years. These interpolated curves were used solely to provide a graphical representation of the empirical percentiles observed at ages 4, 5, and 6 years and should not be interpreted as continuously age-modeled reference curves.

To assess the precision of the age- and sex-specific percentile estimates, 95% confidence intervals were obtained using non-parametric bootstrap resampling within each age-by-sex subgroup. A total of 10,000 bootstrap samples were generated by resampling participants with replacement, and the same empirical percentile-estimation procedure was applied to each bootstrap sample. Percentile-based 95% confidence intervals were defined by the 2.5th and 97.5th percentiles of the corresponding bootstrap distributions. A fixed random seed was used to ensure reproducibility.

Associations between maximum HGS and age and anthropometric characteristics (body weight, height, body mass index, and waist circumference) were examined using Spearman’s rank correlation coefficients (ρ). Correlation coefficients were interpreted as negligible (0.00–0.10), weak (0.10–0.39), moderate (0.40–0.69), strong (0.70–0.89), and very strong (0.90–1.00) according to Schober et al. [17].

For graphical presentation of the anthropometric relationships, individual observations were plotted separately by sex and descriptive sex-specific ordinary least-squares fitted lines with 95% confidence intervals were superimposed. These fitted lines were used solely for visualization and were not interpreted as adjusted or causal associations.

To examine the independent associations of age, sex, and anthropometric characteristics with maximum HGS, multiple linear regression analyses were performed. The primary model included age, sex, height, body weight, waist circumference, and study year as predictors. Because BMI is mathematically derived from body weight and height, it was examined in a separate model including age, sex, BMI, waist circumference, and study year. Standardized and unstandardized regression coefficients with 95% confidence intervals were reported. Multicollinearity was assessed using variance inflation factors and tolerance values. Hierarchical regression models were additionally used to quantify the incremental variance in maximum HGS explained by height and by body weight and waist circumference beyond age, sex, and study year. Sex-by-anthropometric-variable interaction terms were examined separately to determine whether these associations differed between boys and girls.

To evaluate the appropriateness of pooling data collected in 2022 and 2025, additional cohort-comparability and sensitivity analyses were performed. Participant characteristics and maximum HGS were compared between cohorts, including age- and sex-stratified comparisons. In addition, multiple linear regression models examined whether study year was associated with maximum HGS after adjustment for age, sex, and height. Study year-by-age and study year-by-sex interaction terms were examined separately to assess whether age- or sex-related patterns differed between cohorts. Finally, the main model coefficients obtained with and without adjustment for study year were compared to evaluate the influence of pooling the two datasets.

All statistical tests were two-sided, and statistical significance was established at *p* < 0.05.

## 3. Results

A total of 899 preschool children (464 boys and 435 girls) were included in the final analyses. The mean age of the participants was approximately 5.3 years in both sexes. Boys presented slightly higher mean values than girls for body weight, height, waist circumference, and HGS in both hands. Maximum HGS averaged 8.91 ± 2.27 kg in boys and 7.98 ± 2.15 kg in girls. The descriptive characteristics of the study population are presented in Table 1.

To assess the appropriateness of pooling data from the two collection periods, sensitivity analyses were conducted to evaluate the comparability of the 2022 and 2025 cohorts. The final analytical sample comprised 553 participants from 2022 and 346 from 2025. Although modest differences were observed in age distribution, height, BMI, and left-hand HGS, maximum HGS did not differ significantly between cohorts (8.44 ± 2.30 vs. 8.59 ± 2.49 kg; mean difference = 0.16 kg, 95% CI −0.17 to 0.48; *p* = 0.350; Cohen’s d = 0.07). After adjustment for age, sex, and height, study year was not independently associated with maximum HGS (B = −0.05 kg, 95% CI −0.32 to 0.21; *p* = 0.687). Neither the study year × age interaction (*p* = 0.950) nor the study year × sex interaction (*p* = 0.398) was statistically significant. Furthermore, adjustment for study year produced only negligible changes in the age, sex, and height coefficients (<1.2%), supporting the pooling of both cohorts for the primary analyses (Supplementary Table S2).

Boys showed higher mean maximum HGS than girls at all three ages, and the age-specific sex differences were statistically significant (Table 2). The mean difference (boys − girls) was 1.14 kg at age 4 (95% CI: 0.57–1.71; *p* < 0.001; Cohen’s d = 0.65, 95% CI: 0.33–0.98), 0.59 kg at age 5 (95% CI: 0.14–1.05; *p* = 0.011; Cohen’s d = 0.28, 95% CI: 0.06–0.50), and 1.00 kg at age 6 (95% CI: 0.58–1.42; *p* < 0.001; Cohen’s d = 0.46, 95% CI: 0.26–0.65). In the complementary age-adjusted analysis, boys also had higher maximum HGS than girls across the full sample (B = 0.88 kg, 95% CI: 0.61–1.16; *p* < 0.001).

**Figure 1:**
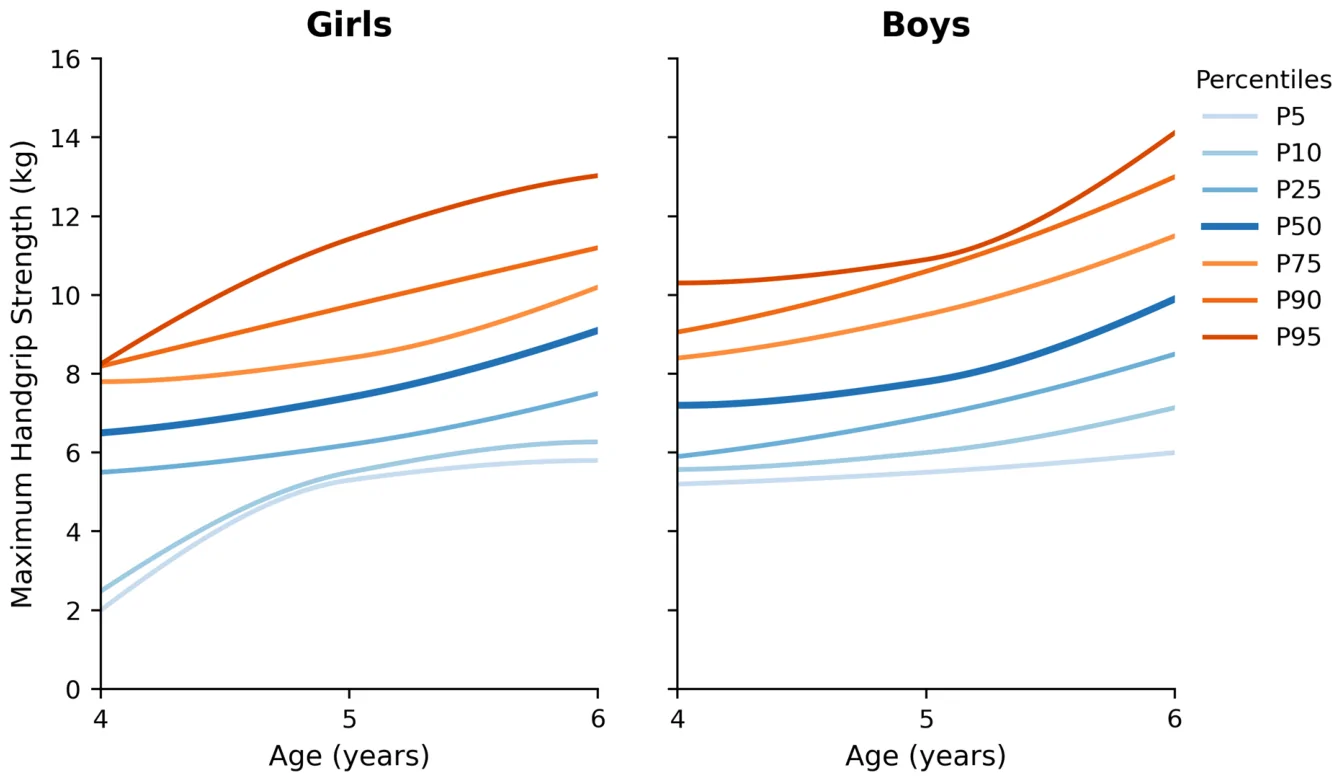
Interpolated age- and sex-specific percentile curves for maximum handgrip strength in preschool children from the Araucanía Region, Chile. Note: Curves represent shape-preserving piecewise cubic Hermite interpolation (PCHIP) through the empirical percentile values observed at ages 4, 5, and 6 years and are provided for graphical presentation only; they should not be interpreted as continuously age-modeled reference curves.

Age- and sex-specific percentile reference values for maximum HGS are presented in Table 3, while the corresponding interpolated percentile curves are shown in Figure 1. Percentile values increased progressively with age in both sexes, reflecting the expected developmental improvements in muscular strength during the preschool years. For example, the median (P50) maximum HGS increased from 6.5 to 9.1 kg in girls and from 7.2 to 9.9 kg in boys between 4 and 6 years of age. Across all age groups, boys exhibited higher percentile values than girls, particularly at the upper percentiles (P75–P95), indicating greater maximum HGS throughout the distribution. Figure 1 illustrates the interpolated age- and sex-specific percentile curves for maximum HGS. The progressive upward displacement of the percentile curves from 4 to 6 years reflects the observed increase in muscular strength across the three age categories, whereas the generally higher curves observed in boys indicate greater maximum HGS across most of the distribution.

Age- and sex-specific percentile reference values for right- and left-handgrip strength are provided in Supplementary Table S3. These supplementary data complement the main reference values by allowing separate evaluation of each hand when methodologically or scientifically required.

Bootstrap analyses were additionally performed to quantify the uncertainty of the age- and sex-specific percentile estimates (Supplementary Table S4). Uncertainty varied across percentiles and age–sex groups and was particularly notable for the lower tail among 4-year-old girls. In this subgroup, the P5 estimate was 2.00 kg (95% bootstrap CI 2.00–5.00 kg), whereas P10 and P25 were 2.48 and 5.50 kg, respectively. Targeted review of the underlying observations confirmed that the marked P10–P25 separation reflected repeated clusters of low recorded HGS values rather than an isolated outlier or identifiable data-entry error.

Maximum HGS was positively associated with age and all anthropometric variables examined (Table 4). Height showed the strongest correlation with maximum HGS (ρ = 0.554, *p* < 0.001), followed by body weight (ρ = 0.463, *p* < 0.001) and age (ρ = 0.458, *p* < 0.001). Waist circumference and body mass index demonstrated a weak positive association with maximum HGS (ρ = 0.307 and ρ = 0.184, respectively; both *p* < 0.001).

**Figure 2:**
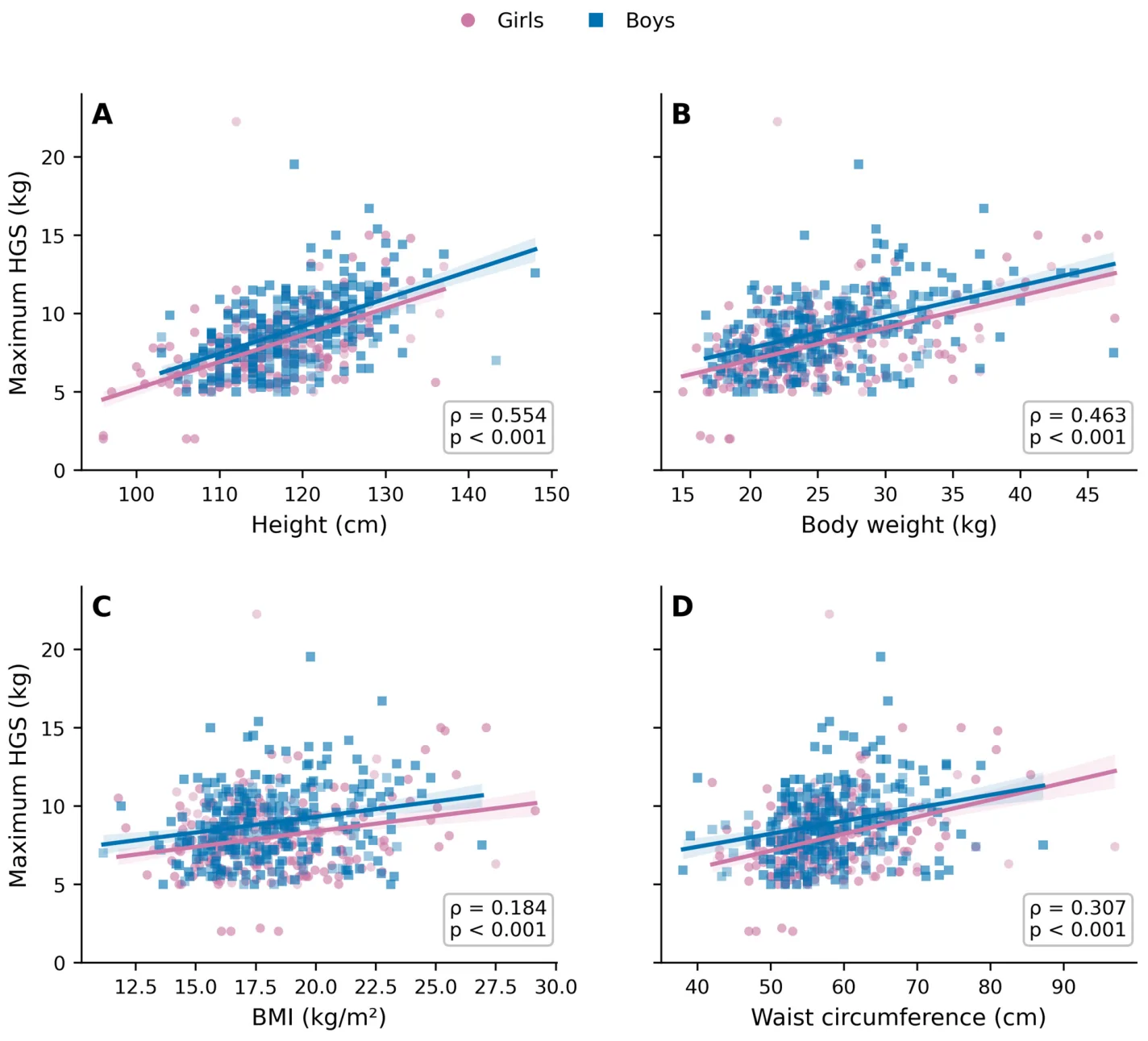
Relationships between maximum handgrip strength and anthropometric characteristics in preschool children from the Araucanía Region, Chile. Individual points represent participants, with girls shown as circles and boys as squares. Solid lines represent descriptive sex-specific linear fitted trends, with shaded areas indicating 95% confidence intervals. Panels show the observed relationships between maximum handgrip strength and (**A**) height, (**B**) body weight, (**C**) body mass index, and (**D**) waist circumference. Values displayed within each panel represent pooled Spearman rank correlation coefficients (ρ) and two-sided *p*-values. These unadjusted relationships are descriptive and should not be interpreted as independent or causal associations.

Figure 2 illustrates the observed relationships between maximum HGS and anthropometric characteristics. Maximum HGS showed the clearest positive relationship with height (ρ = 0.554, *p* < 0.001), followed by body weight (ρ = 0.463, *p* < 0.001) and waist circumference (ρ = 0.307, *p* < 0.001), whereas the association with BMI was comparatively weaker (ρ = 0.184, *p* < 0.001). The sex-specific fitted lines provide a descriptive visualization of these relationships and should not be interpreted as adjusted or causal effects.

In multivariable analysis, age, sex, height, and body weight remained independently and positively associated with maximum HGS (Table 5). Height showed the strongest standardized association (β = 0.306, *p* < 0.001), followed by body weight (β = 0.256, *p* < 0.001), whereas study year was not independently associated with maximum HGS (β = 0.020, *p* = 0.481). Waist circumference showed a small inverse association in the primary model (β = −0.088, *p* = 0.043). Overall, the model explained 36.1% of the variance in maximum HGS (R^2^ = 0.361; adjusted R^2^ = 0.356). In hierarchical analyses, adding height to a model including age, sex, and study year increased the explained variance by 12.0% (*p* < 0.001), whereas the subsequent addition of body weight and waist circumference accounted for an additional 1.8% (*p* < 0.001). In the alternative model including BMI instead of height and body weight, BMI was not independently associated with maximum HGS (*p* = 0.154). Furthermore, none of the sex-by-anthropometric-variable interactions was statistically significant (all *p* ≥ 0.444), providing no evidence that these associations differed between boys and girls (Supplementary Table S5).

## 4. Discussion

The present study provides updated age- and sex-specific reference values for maximum HGS in preschool children aged 4–6 years from the Araucanía Region, Chile, using a large regional convenience sample assessed under standardized conditions. Four principal findings emerged. First, maximum HGS increased progressively with age in both boys and girls. Second, boys demonstrated significantly higher maximum HGS than girls at all three ages, although the magnitude of the sex difference varied across age groups. Third, age- and sex-specific empirical percentiles and interpolated percentile curves were established, providing regional reference information for children assessed under comparable conditions. Finally, maximum HGS was positively associated with anthropometric characteristics, with height and body weight showing the most consistent associations in the multivariable analyses. Collectively, these findings provide updated regional reference information for characterizing muscular fitness during the preschool years.

The age- and sex-related patterns observed in the present study are consistent with previous reference studies conducted in preschool and pediatric populations [3,4,5,8,10]. Recent evidence from Spain similarly reported a progressive increase in HGS throughout childhood together with consistently higher values in boys than in girls, although sex differences during the preschool years remained relatively small [7]. Comparable developmental trajectories have also been described in European preschool children, supporting the concept that muscular strength follows a predictable biological pattern during early childhood despite differences in geographical location, ethnicity, and study design [18]. This consistency across diverse populations reinforces the biological validity of the age-related pattern observed in the present study while emphasizing the importance of establishing context-specific reference values for contextualized interpretation of muscular fitness.

Although the overall developmental pattern observed in our study agrees with previous investigations, direct comparisons of absolute reference values should be interpreted with caution. Differences in sampling strategies, age categorization, socioeconomic characteristics, anthropometric profiles, dynamometer models, testing protocols, and the operational definition of HGS may substantially influence reference values reported across studies [7,18]. In the present study, maximum HGS was defined as the single highest valid value obtained across trials from either hand, thereby representing each child’s maximal observed grip-strength capacity irrespective of hand dominance. Other studies have reported dominant-hand strength, right-hand strength, the best value for each hand separately, or bilateral mean values. These approaches are not directly interchangeable, and selecting the highest value across both hands and trials may result in somewhat higher estimates than approaches based on a predetermined hand or averaged measurements. Therefore, comparisons between the present percentiles and previously published reference values should account for differences in HGS outcome definition and testing protocol. To facilitate comparisons with studies using side-specific outcomes, age- and sex-specific percentile values for right- and left-hand HGS are additionally provided in the Supplementary Materials.

The present study also expands previous Chilean evidence by providing updated reference values derived from a large regional sample of preschool children. Compared with previously published Chilean reference data, the present investigation includes a substantially larger sample, standardized assessment procedures based on the PREFIT protocol, empirical percentile distributions, and interpolated percentile curves, thereby providing additional reference information for the assessment and interpretation of muscular fitness in preschool children [7,11].

The progressive increase in maximum HGS between 4 and 6 years of age is consistent with the rapid neuromuscular, musculoskeletal, and motor development characteristic of the preschool period [19,20]. Improvements in motor coordination, neuromuscular function, and movement efficiency, together with physical growth and maturation, likely contribute to the greater force production observed with increasing age [19,20].

Beyond age-related increases in muscular strength, emerging evidence suggests that HGS during early childhood may reflect broader aspects of physical growth and development. Reference data from pediatric populations indicate that HGS is closely related to age, body size, and nutritional characteristics [7]. Importantly, studies conducted at very young ages have also reported associations between lower HGS and less favorable motor and developmental outcomes, suggesting that grip strength may capture aspects of neuromuscular and functional development beyond body size alone [21]. In kindergarten-aged children, longitudinal observations further indicate that HGS changes across early childhood and may differ according to patterns of physically active play [22]. These findings support the interpretation of HGS as a practical indicator of muscular fitness and physical development during the preschool years. However, because the available evidence remains limited and heterogeneous, particularly in children younger than seven years, HGS should not be interpreted as an isolated marker of developmental or health status, and further longitudinal studies are needed to clarify its relationship with motor, behavioral, and health trajectories.

Although boys consistently demonstrated higher maximum HGS than girls, the magnitude of these differences remained relatively small, which is consistent with previous studies conducted in preschool populations [7,18]. Before puberty, sex-related differences in muscular strength are generally attributed to subtle variations in body size, habitual physical activity, motor experiences, and neuromuscular development rather than to hormonal influences [23,24]. Consequently, although sex differences are modest during early childhood, the use of sex-specific reference values may improve the accuracy of muscular fitness assessment and facilitate more appropriate interpretation of children’s physical development.

Beyond providing updated descriptive data, the present age- and sex-specific percentiles offer a practical framework for contextualizing maximum HGS performance among preschool children from the Araucanía Region and populations assessed under comparable conditions. Specifically, an individual HGS measurement can be positioned relative to the observed distribution for children of the same age and sex, allowing researchers and professionals involved in pediatric physical fitness assessment to describe whether performance falls within the lower, middle, or upper portion of the regional reference distribution. The percentile tables and interpolated curves may also facilitate comparisons across studies conducted using comparable HGS protocols and provide descriptive reference points for HGS measurements obtained during follow-up or physical activity and fitness intervention studies [1,24,25]. However, because these percentiles were derived from a regional convenience sample using cross-sectional data and were not validated against health or developmental outcomes, they should not be interpreted as clinical cut-offs, diagnostic thresholds, or validated thresholds for evaluating individual longitudinal change.

The associations between maximum HGS and anthropometric characteristics provide further evidence of the relationship between physical growth and muscular strength during early childhood [1,26]. Although the bivariate analyses showed positive correlations between maximum HGS and all anthropometric indicators examined, the multivariable analyses provided a more nuanced interpretation. Height remained independently associated with maximum HGS after adjustment for age, sex, body weight, waist circumference, and study year and showed the largest standardized association among the anthropometric predictors. Hierarchical analyses further demonstrated that the addition of height explained a substantial proportion of variance in maximum HGS beyond age, sex, and study year. Body weight also retained an independent positive association, although its additional contribution beyond height was comparatively smaller. These findings are consistent with previous evidence indicating that linear growth and increasing body size are closely related to muscular strength development during childhood, reflecting progressive musculoskeletal development and increasing functional capacity [1,26].

In contrast, the associations involving BMI and waist circumference were less consistent after multivariable adjustment. BMI, which showed only a weak positive bivariate correlation with maximum HGS, was not independently associated with HGS in the alternative adjusted model. One possible explanation for this finding is the limited ability of BMI to distinguish fat mass from fat-free mass, the latter being more directly related to force production [27]. Similarly, although waist circumference was positively correlated with maximum HGS in the bivariate analysis, its adjusted association was small and varied according to model specification, suggesting that its relationship with muscular strength may largely reflect shared variation with overall body size rather than an independent association. Collectively, these findings indicate that linear growth and body size, particularly height and body weight, are more consistently associated with maximum HGS during the preschool years than BMI or central anthropometric measures. Furthermore, the absence of significant sex-by-anthropometric-variable interactions suggests that these associations were broadly comparable between boys and girls.

The present study has several strengths that should be acknowledged. First, it provides updated age- and sex-specific reference values derived from one of the largest regional samples of Chilean preschool children currently available. Second, all assessments were conducted using standardized procedures based on the PREFIT protocol and the same model of handgrip dynamometer, thereby minimizing methodological variability. Third, percentile distributions and interpolated percentile curves were generated separately for boys and girls, facilitating the interpretation of HGS performance and future epidemiological comparisons. Finally, the combination of bivariate and multivariable analyses provided additional insight into the independent statistical associations between physical growth, anthropometric characteristics, and muscular strength during early childhood.

Several limitations should also be considered. First, the cross-sectional design precludes evaluation of individual developmental trajectories or causal relationships. Second, participants were recruited through convenience sampling from educational centers located exclusively in the Araucanía Region; therefore, the sample is not nationally representative of Chilean preschool children. Accordingly, the present percentiles should be interpreted as regional reference values for children assessed under comparable conditions rather than as national reference values for all Chilean preschool children. Differences in demographic, socioeconomic, environmental, and lifestyle characteristics across Chilean regions may limit the generalizability of these findings to the broader national population. Third, participants were recruited from multiple educational centers, and the potential clustering of children within centers was not explicitly accounted for in the statistical analyses. Children attending the same educational center may share environmental, socioeconomic, or educational characteristics, resulting in some degree of within-center correlation. Consequently, the assumption of independence between observations may have affected the precision of some estimates. Future multicenter studies should account for the hierarchical structure of the data using appropriate multilevel or cluster-robust analytical approaches. Fourth, data were pooled from two independent collection periods conducted in 2022 and 2025, which could potentially introduce temporal or cohort-related variability. However, sensitivity analyses showed no independent association between study year and maximum HGS after adjustment for age, sex, and height, no significant study year-by-age or study year-by-sex interactions, and negligible changes in the principal model coefficients after adjustment for study year. These findings support the comparability of the two cohorts, although residual unmeasured cohort effects cannot be completely excluded. In addition, chronological age was available only in completed years rather than in months or decimal years. Consequently, the age-specific percentiles were based on three discrete age categories (4, 5, and 6 years), and the graphical curves represent interpolation between the empirical percentiles at these ages rather than continuous age-based percentile modeling. This reduced age resolution may limit the precision with which developmental changes in HGS can be characterized during this period of rapid growth. Future studies should record exact dates of birth and assessment to enable continuous age modeling and the development of more precise reference curves. The precision of the empirical percentile estimates also varied across age–sex groups, particularly at the distribution tails. Bootstrap analyses indicated substantial uncertainty for some extreme percentile estimates, most notably the P5 among 4-year-old girls; therefore, extreme percentiles should be interpreted with greater caution than central percentile estimates. Another methodological consideration is that the use of the single highest HGS value obtained across either hand as the primary outcome may limit direct comparability with studies reporting dominant-hand, right-hand, or averaged bilateral HGS values. Finally, although the multivariable analyses accounted for several relevant growth and anthropometric characteristics, information on biological maturation, habitual physical activity, nutritional status, and socioeconomic characteristics was not available; therefore, residual confounding by these unmeasured biological, behavioral, and socioeconomic factors cannot be excluded. Future multicenter longitudinal studies including nationally representative samples and a broader range of biological and behavioral variables are warranted to confirm and extend the present findings.

## 5. Conclusions

This study provides updated age- and sex-specific regional reference values for maximum HGS in preschool children aged 4–6 years from the Araucanía Region, Chile, based on a large regional convenience sample and standardized assessment procedures. Maximum HGS increased with age, was significantly higher in boys than in girls at each age and was most consistently associated with height and body weight. The resulting percentile tables and interpolated curves provide a practical framework for contextualizing HGS performance relative to children of the same age and sex assessed under comparable conditions. However, these findings should be regarded as preliminary regional reference data rather than nationally representative normative values, clinical cut-offs, or diagnostic thresholds. Independent validation in probability-based, nationally representative, and longitudinal samples is needed before broader population-level or clinical applications can be considered.

## Figures and Tables

**Table 1:** Characteristics of the Study Participants by Sex.

Variable	Total (*n* = 899)	Girls (*n* = 435)	Boys (*n* = 464)
Age (years)	5.30 ± 0.74	5.28 ± 0.73	5.31 ± 0.74
Weight (kg)	25.29 ± 5.50	24.83 ± 5.54	25.72 ± 5.42
Height (cm)	117.44 ± 7.11	116.46 ± 7.30	118.36 ± 6.81
BMI (kg/m^2^)	18.17 ± 2.65	18.17 ± 2.75	18.16 ± 2.54
Waist Circumference (cm)	58.33 ± 6.90	57.88 ± 6.94	58.74 ± 6.83
Right Handgrip (kg)	7.90 ± 2.22	7.48 ± 2.17	8.27 ± 2.18
Left Handgrip (kg)	7.82 ± 2.30	7.35 ± 2.12	8.24 ± 2.37
Maximum Handgrip Strength (kg)	8.46 ± 2.27	7.98 ± 2.15	8.91 ± 2.27

Data are presented as mean ± standard deviation (SD).

**Table 2:** Handgrip Strength According to Age and Sex in Preschool Children from the Araucanía Region, Chile.

Age (Years)	Sex	Right Handgrip, kg	Left Handgrip, kg	Maximum Handgrip Strength, kg
4	Girls	5.97 ± 1.93 (*n* = 71)	5.95 ± 1.85 (*n* = 65)	6.27 ± 1.89 (*n* = 72)
	Boys	6.95 ± 1.48 (*n* = 77)	6.83 ± 1.56 (*n* = 78)	7.41 ± 1.59 (*n* = 78)
	Sex comparison †			1.14 (0.57–1.71); *p* < 0.001; d = 0.65 (0.33–0.98)
5	Girls	7.12 ± 2.08 (*n* = 165)	6.80 ± 1.93 (*n* = 160)	7.63 ± 2.07 (*n* = 165)
	Boys	7.50 ± 1.74 (*n* = 161)	7.45 ± 2.45 (*n* = 159)	8.22 ± 2.13 (*n* = 161)
	Sex comparison †			0.59 (0.14–1.05); *p* = 0.011; d = 0.28 (0.06–0.50)
6	Girls	8.42 ± 2.24 (*n* = 196)	8.24 ± 2.04 (*n* = 197)	8.99 ± 2.10 (*n* = 198)
	Boys	9.30 ± 2.23 (*n* = 225)	9.36 ± 2.30 (*n* = 223)	9.99 ± 2.30 (*n* = 225)
	Sex comparison †			1.00 (0.58–1.42); *p* < 0.001; d = 0.46 (0.26–0.65)

Handgrip strength values are presented as mean ± standard deviation (SD), with the number of valid observations (*n*) shown for each measure. † Age-specific sex comparisons refer to maximum HGS and were performed using two-sided Welch independent-samples *t*-tests. Mean differences are expressed as boys minus girls and are presented as mean difference (95% confidence interval). Cohen’s d values with 95% confidence intervals are also reported, with positive values indicating higher maximum HGS in boys. HGS, handgrip strength.

**Table 3:** Age- and Sex-Specific Percentile Reference Values for Maximum Handgrip Strength in Preschool Children from the Araucanía Region, Chile.

Age	Sex	*n*	P5	P10	P25	P50	P75	P90	P95
4	Girls	72	2.00 *	2.48	5.50	6.50	7.80	8.19	8.25
	Boys	78	5.20	5.57	5.90	7.20	8.40	9.06	10.31
5	Girls	165	5.30	5.50	6.20	7.40	8.40	9.72	11.42
	Boys	161	5.50	6.00	6.90	7.80	9.50	10.60	10.90
6	Girls	198	5.80	6.27	7.50	9.10	10.20	11.20	13.03
	Boys	225	6.00	7.14	8.50	9.90	11.50	13.00	14.12

Values are expressed in kilograms (kg). P5, P10, P25, P50, P75, P90, and P95 represent the 5th, 10th, 25th, 50th, 75th, 90th, and 95th percentiles, respectively. * The P5 estimate for 4-year-old girls showed considerable uncertainty (95% bootstrap CI: 2.00–5.00 kg) and should therefore be interpreted with caution. Bootstrap 95% confidence intervals for all percentile estimates are provided in Supplementary Table S4.

**Table 4:** Correlations of Maximum Handgrip Strength with Age and Anthropometric Characteristics in Preschool Children from the Araucanía Region, Chile.

Variable	Spearman’s ρ	*p*-Value
Age	0.458	<0.001
Weight	0.463	<0.001
Height	0.554	<0.001
BMI	0.184	<0.001
Waist Circumference	0.307	<0.001

Spearman’s rank correlation coefficients (ρ) were calculated between maximum handgrip strength and age and anthropometric characteristics. Statistical significance was set at *p* < 0.05. BMI, body mass index.

**Table 5:** Multivariable linear regression analysis of factors associated with maximum handgrip strength in preschool children from the Araucanía Region, Chile.

Predictor	B	95% CI	Standardized β	*p*-Value
Age, years	0.502	0.289 to 0.716	0.157	<0.001
Sex: boys vs. girls	0.604	0.350 to 0.858	0.127	<0.001
Height, cm	0.098	0.070 to 0.126	0.306	<0.001
Body weight, kg	0.109	0.063 to 0.155	0.256	<0.001
Waist circumference, cm	−0.029	−0.056 to −0.001	−0.088	0.043
Study year: 2025 vs. 2022	0.096	−0.171 to 0.362	0.020	0.481

B represents the unstandardized regression coefficient. The model included age, sex, height, body weight, waist circumference, and study year simultaneously. Girls and the 2022 cohort were the reference categories for sex and study year, respectively. Model *n* = 894; R^2^ = 0.361; adjusted R^2^ = 0.356; overall model *p* < 0.001. β, standardized regression coefficient; CI, confidence interval; HGS, handgrip strength. The multivariable model included 894 complete cases because five participants had missing data for one or more anthropometric variables included in the model. No missing values were imputed.

## Data Availability

The data presented in this study are available on request from the corresponding author. The data are not publicly available due to ethical standards.

## Supplementary Materials

The following supporting information can be downloaded at https://www.mdpi.com/article/10.3390/children13091200/s1, Table S1: Distribution of participants by study cohort, municipality, and educational center; Table S2: Comparability and sensitivity analyses between the 2022 and 2025 study cohorts; Table S3: Age- and sex-specific percentile reference values for right and left handgrip strength in preschool children from the Araucanía Region, Chile; Table S4: Bootstrap 95% confidence intervals for age- and sex-specific percentile estimates of maximum handgrip strength; Table S5: Additional multivariable, hierarchical, interaction, and sensitivity analyses of maximum handgrip strength.

## Abbreviations

The following abbreviations are used in this manuscript:

BMI	Body Mass Index
CI	Confidence Interval
HGS	Handgrip Strength
PREFIT	assessing FITness in PREschoolers
