# Supplementary material for: Updated Age- and Sex-Specific Reference Values and Anthropometric Correlates of Maximum Handgrip Strength in Preschool Children from the Araucanía Region, Chile
Source: Children (Basel). 2026 Sep 5;13(9):1200. doi: 10.3390/children13091200 (PMC13604991; doi:10.3390/children13091200)
Supplement: Supplementary file 1 [file children-13-01200-s001.zip › children-4452358-supplementary.pdf]

Table S1. Distribution of participants by study cohort, municipality, and educational center.

Study cohort	Municipality	Educational center	Initial n
**2022**	Pucón	Educational center 1	32
	Cunco	Educational center 2	41
	Victoria	Educational center 3	46
	Melipeuco	Educational center 4	41
	Loncoche	Educational center 5	73
	Angol	Educational center 6	64
	Temuco	Educational center 7	92
	Lautaro	Educational center 8	42
	Chol Chol	Educational center 9	39
	Curacautín	Educational center 10	37
	Collipulli	Educational center 11	25
	Villarrica	Educational center 12	35
**2022 total**	**12 municipalities**	**12 educational centers**	**567**
**2025**	Temuco	Educational center 1	22
	Temuco	Educational center 2	10
	Temuco	Educational center 3	36
	Temuco	Educational center 4	67
	Temuco	Educational center 5	30
	Temuco	Educational center 6	34
	Temuco	Educational center 7	62
	Temuco	Educational center 8	30
	Temuco	Educational center 9	35
	Temuco	Educational center 10	39
**2025 total**	**1 municipality**	**10 educational centers**	**365**
**Overall**	—	**22 educational centers**	**932**

Values represent the number of participants initially contributed by each educational center before application of the final analytical eligibility and data-quality criteria. In 2022, one educational center participated from each of the 12 municipalities. In 2025, all 10 participating educational centers were in Temuco. Educational centers are identified numerically to avoid unnecessary disclosure of institutional identities.

Table S2. Comparability and sensitivity analyses between the 2022 and 2025 study cohorts

Panel A. Characteristics of the 2022 and 2025 cohorts

Characteristic	2022	2025	Difference (2025 − 2022)	p-value	Effect size
Analytical sample, n	553	346	—	—	—
Girls, n (%)	280 (50.6)	155 (44.8)	—	0.102	V = 0.055
Boys, n (%)	273 (49.4)	191 (55.2)	—	0.102	V = 0.055
Age 4 years, n (%)	94 (17.0)	56 (16.2)	—	0.004	V = 0.111
Age 5 years, n (%)	178 (32.2)	148 (42.8)	—	0.004	V = 0.111
Age 6 years, n (%)	281 (50.8)	142 (41.0)	—	0.004	V = 0.111
Age, years	5.34 ± 0.75	5.25 ± 0.72	−0.09	0.074	d = −0.12
Body weight, kg	25.55 ± 5.54 (n = 551)	24.93 ± 5.65 (n = 346)	−0.61	0.111	d = −0.11
Height, cm	116.94 ± 7.50	118.51 ± 7.22	+1.57	0.002	d = 0.21
BMI, kg/m²	18.54 ± 2.68 (n = 551)	17.60 ± 2.70 (n = 346)	−0.93	<0.001	d = −0.35
Waist circumference, cm	58.24 ± 7.00 (n = 550)	58.43 ± 7.82 (n = 346)	+0.19	0.709	d = 0.03
Right HGS, kg	7.93 ± 2.28 (n = 550)	7.90 ± 2.29 (n = 345)	−0.03	0.837	d = −0.01
Left HGS, kg	7.65 ± 2.33 (n = 549)	8.11 ± 2.44 (n = 333)	+0.46	0.006	d = 0.19
Maximum HGS, kg	8.44 ± 2.30	8.59 ± 2.49	+0.16	0.350	d = 0.07

Panel B. Multivariable linear regression model for maximum HGS

**Predictor**	**B**	**95% CI**	**Standardized β**	**p-value**
Study year: 2025 vs. 2022	−0.05	−0.32 to 0.21	−0.01	0.687
Age, years	0.53	0.32 to 0.75	0.17	<0.001
Sex: boys vs. girls	0.61	0.35 to 0.87	0.13	<0.001
Height, cm	0.14	0.12 to 0.16	0.44	<0.001

Panel C. Study-year interaction and pooling sensitivity analyses

Analysis	Estimate	95% CI	p-value / change
Study year × age	B = −0.01 kg/year	−0.37 to 0.35	p = 0.950
Study year × sex	B = 0.23 kg	−0.30 to 0.75	p = 0.398
Age coefficient: model without vs. with study year	0.541 vs. 0.535	—	1.2% change
Sex coefficient: model without vs. with study year	0.608 vs. 0.610	—	0.3% change
Height coefficient: model without vs. with study year	0.141 vs. 0.142	—	0.5% change

Values are presented as mean ± SD or n (%), as appropriate. Differences for continuous variables were calculated as 2025 minus 2022 and assessed using Welch's t-test; effect sizes are expressed as Cohen's d. Sex and age-category distributions were compared using chi-square tests, with Cramér's V as the corresponding effect-size measure; p-values and Cramér's V refer to the overall distribution and are therefore repeated across category rows. In the multivariable model, 2022 and girls were the reference categories. Interaction terms were examined in separate models adjusted for the corresponding main effects, age, sex, and height. Relative coefficient change represents the absolute percentage change after inclusion of study year in the adjusted model. HGS, handgrip strength; BMI, body mass index; CI, confidence interval; SD, standard deviation.

Table S3. Age- and Sex-Specific Percentile Reference Values for Right and Left Handgrip Strength in Preschool Children from the Araucanía Region, Chile.

Age	Sex	n	P5	P10	P25	P50	P75	P90	P95
Right Handgrip									
4	Girls	71	2.2	2.50	5.30	6.10	7.25	7.80	8.10
	Boys	77	5.16	5.26	5.50	7.10	8.00	8.74	9.08
5	Girls	164	5.00	5.13	5.60	6.70	7.90	9.10	9.97
	Boys	161	5.00	5.30	5.90	7.50	8.80	9.90	10.20
6	Girls	196	5.40	5.80	6.50	8.40	9.70	10.95	13.05
	Boys	225	5.60	6.54	7.50	9.30	10.60	12.40	13.00
Left Handgrip									
4	Girls	65	2.00	2.20	5.30	6.00	7.10	8.10	8.28
	Boys	78	5.00	5.27	5.80	6.50	7.55	8.86	10,30
5	Girls	160	5.00	5.00	5.60	6.60	8.10	9.00	9.90
	Boys	159	5.00	5.28	6.00	7.20	8.80	10.50	10.90
6	Girls	197	5.50	5.80	6.80	8.00	9.40	11.10	11.70
	Boys	223	5.80	6.50	7.65	9.40	10.80	11.78	13.78

Table S4. Bootstrap 95% Confidence Intervals for Age- and Sex-Specific Percentile Estimates of Maximum Handgrip Strength.

Sex	Age (years)	n	P5	P10	P25	P50	P75	P90	P95
Girls	4	72	2.00 (2.00–5.00)	2.48 (2.00–5.03)	5.50 (5.00–6.10)	6.50 (6.10–6.80)	7.80 (7.10–7.90)	8.19 (7.80–8.29)	8.25 (8.05–10.20)
	5	165	5.30 (5.20–5.50)	5.50 (5.30–5.60)	6.20 (5.90–6.60)	7.40 (7.10–7.70)	8.40 (8.20–8.80)	9.72 (9.10–10.00)	11.42 (9.80–12.00)
	6	198	5.80 (5.50–6.10)	6.27 (5.80–6.80)	7.50 (7.10–7.80)	9.10 (8.80–9.35)	10.20 (9.90–10.48)	11.20 (10.90–12.10)	13.03 (11.50–14.80)
Boys	4	78	5.20 (5.00–5.60)	5.57 (5.20–5.80)	5.90 (5.80–6.60)	7.20 (6.60–7.80)	8.40 (8.00–8.80)	9.06 (8.80–10.41)	10.31 (9.00–11.50)
	5	161	5.50 (5.30–5.90)	6.00 (5.50–6.30)	6.90 (6.50–7.20)	7.80 (7.50–8.10)	9.50 (9.00–9.70)	10.60 (10.00–10.90)	10.90 (10.60–11.80)
	6	225	6.00 (5.60–6.82)	7.14 (6.50–7.50)	8.50 (8.20–8.80)	9.90 (9.60–10.30)	11.50 (11.10–11.70)	13.00 (12.30–13.80)	14.12 (13.40–14.50)

Values are expressed in kilograms as empirical percentile estimate (95% bootstrap confidence interval). Empirical percentiles were estimated separately within each age-by-sex subgroup using pandas.Series.quantile() with linear interpolation (interpolation='linear'). Confidence intervals were obtained using non-parametric bootstrap resampling with replacement within each age-by-sex subgroup (10,000 replications). Percentile-based 95% confidence intervals correspond to the 2.5th and 97.5th percentiles of the bootstrap distributions. A fixed random seed was used to ensure reproducibility. HGS, handgrip strength; CI, confidence interval.

Table S5. Additional multivariable, hierarchical, interaction, and sensitivity analyses of maximum handgrip strength.

Analysis / Predictor	n	B / R^2	95% CI / Adjusted R^2	Standardized β / ΔR^2	F-change	p-value
**Panel A. Alternative multivariable model including BMI**						
Age, years	894	1.218	1.028 to 1.407	0.379	—	<0.001
Sex: boys vs. girls	894	0.842	0.572 to 1.112	0.177	—	<0.001
BMI, kg/m²	894	0.052	−0.019 to 0.123	0.059	—	0.154
Waist circumference, cm	894	0.050	0.023 to 0.077	0.154	—	<0.001
Study year: 2025 vs. 2022	894	0.273	−0.012 to 0.558	0.056	—	0.061
**Model summary**	**894**	**R^2 = 0.260**	**Adjusted R^2 = 0.255**	—	—	**<0.001**
**Panel B. Hierarchical regression analysis of maximum HGS**						
Model 1: Age + sex + study year	894	R^2 = 0.222	Adjusted R^2 = 0.220	—	—	—
Model 2: Model 1 + height	894	R^2 = 0.342	Adjusted R^2 = 0.340	ΔR^2 = 0.120	162.42	<0.001
Model 3: Model 2 + body weight + waist circumference	894	R^2 = 0.360	Adjusted R^2 = 0.356	ΔR^2 = 0.018	12.46	<0.001
**Panel C. Sex-by-anthropometric-variable interaction analyses**						
Sex × height	899	0.005	−0.029 to 0.040	—	—	0.771
Sex × body weight	897	−0.004	−0.051 to 0.042	—	—	0.849
Sex × waist circumference	896	−0.014	−0.051 to 0.023	—	—	0.444
Sex × BMI	897	−0.010	−0.110 to 0.090	—	—	0.846
**Panel D. Sensitivity analysis for the influential HGS observation**						
Age, years	—	Primary B = 0.502	Sensitivity B = 0.505	Difference = +0.003	—	Primary <0.001; Sensitivity <0.001
Sex: boys vs. girls	—	Primary B = 0.604	Sensitivity B = 0.638	Difference = +0.034	—	Primary <0.001; Sensitivity <0.001
Height, cm	—	Primary B = 0.098	Sensitivity B = 0.098	Difference <0.001	—	Primary <0.001; Sensitivity <0.001
Body weight, kg	—	Primary B = 0.109	Sensitivity B = 0.114	Difference = +0.004	—	Primary <0.001; Sensitivity <0.001
Waist circumference, cm	—	Primary B = −0.029	Sensitivity B = −0.031	Difference = −0.003	—	Primary = 0.043; Sensitivity = 0.022
Study year: 2025 vs. 2022	—	Primary B = 0.096	Sensitivity B = 0.127	Difference = +0.031	—	Primary = 0.481; Sensitivity = 0.331
**Primary model**	**894**	**R^2 = 0.360**	**Adjusted R^2 = 0.356**	—	—	—
**Sensitivity model**	**894**	**R^2 = 0.384**	**Adjusted R^2 = 0.379**	—	—	—

Values are unstandardized regression coefficients (B) unless otherwise indicated. In Panel A, the alternative model included age, sex, BMI, waist circumference, and study year; girls and the 2022 cohort were the reference categories. BMI was examined separately from height and body weight because it is mathematically derived from these variables. Panel B presents hierarchical regression models fitted to the same complete-case sample. ΔR^2 represents the incremental proportion of variance explained after adding the variables specified at each step. In Panel C, each sex interaction was examined in a separate model containing the corresponding main effects and adjustment for age

and study year. In Panel D, the sensitivity analysis treated the influential 22.25-kg unilateral HGS measurement as non-valid and used the valid contralateral measurement (5.5 kg) to calculate maximum HGS. This sensitivity analysis did not materially alter the direction, statistical significance, or substantive interpretation of the primary model. BMI, body mass index; CI, confidence interval; HGS, handgrip strength.
